# Dietary Neuroketotherapeutics for Alzheimer’s Disease: An Evidence Update and the Potential Role for Diet Quality

**DOI:** 10.3390/nu11081910

**Published:** 2019-08-15

**Authors:** Matthew K. Taylor, Russell H. Swerdlow, Debra K. Sullivan

**Affiliations:** 1 Medical Center Department of Dietetics and Nutrition, University of Kansas, Kansas City, KS 66160, USA; 2 Alzheimer’s Disease Center, University of Kansas, Fairway, KS 66205, USA; 3 Medical Center Department of Neurology, University of Kansas, Kansas City, KS 66160, USA

**Keywords:** neuroketotherapeutics, Alzheimer’s disease, cognition, fasting, ketogenic diet, β-hydroxybutyrate, medium-chain triglyceride, bioenergetics, diet quality

## Abstract

Alzheimer’s disease (AD) is a devastating neurodegenerative disease with growing prevalence as the global population ages. Currently available treatments for AD have minimal efficacy and there are no proven treatments for its prodrome, mild cognitive impairment (MCI). AD etiology is not well understood and various hypotheses of disease pathogenesis are currently under investigation. A consistent hallmark in patients with AD is reduced brain glucose utilization; however, evidence suggests that brain ketone metabolism remains unimpaired, thus, there is a great deal of increased interest in the potential value of ketone-inducing therapies for the treatment of AD (neuroketotherapeutics; NKT). The goal of this review was to discuss dietary NKT approaches and mechanisms by which they exert a possible therapeutic benefit, update the evidence available on NKTs in AD and consider a potential role of diet quality in the clinical use of dietary NKTs. Whether NKTs affect AD symptoms through the restoration of bioenergetics, the direct and indirect modulation of antioxidant and inflammation pathways, or both, preliminary positive evidence suggests that further study of dietary NKTs as a disease-modifying treatment in AD is warranted.

## 1. Introduction

Alzheimer’s disease (AD), the most common form of dementia, is a growing problem in the United States and globally. It is predicted that by 2050, nearly 15 million people over the age of 65 years living in the US will have AD, nearly more than tripling the number that the disease currently affects [1]. Coupling this projected growth with an abysmal record of effective therapy development [2] poses a global social and economic threat. The identification of effective disease-modifying therapies (DMT) for AD is dire. The lack of success for many experimental DMTs for AD has been suggested to be due to the administration of therapies too late in the disease progression or misplaced emphasis on downstream hallmarks of the disease, potentially missing the underlying cause. It is, however, recognized that the classic hallmarks of AD, amyloid-β (Aβ) and tau tangles, and modern bioenergetic hallmarks, impaired glucose metabolism and mitochondrial dysfunction, all contribute to AD pathology.

A progressive neurodegenerative disease, AD has been increasingly recognized as a metabolic disease [3,4,5]. The brain requires a substantial energy flux that, under normal conditions, is provided by glucose derived by carbohydrate-rich diets. This energy demand is estimated to be 20% or more of total body energy [6,7]. AD patients consistently exhibit reductions in cerebral glucose utilization [8,9,10,11,12,13,14,15,16,17,18,19,20], which correlates with the severity of cognition impairment [21]. Brain glucose hypometabolism is also seen well before the presence of symptoms and likely contributes to AD encephalopathy [22,23]. Evidence of accompanying peripheral functional metabolism change in AD [3] and reduced brain glucose metabolism in younger adults with increased genetic risks of AD [12,13] suggests that AD-related brain glucose hypometabolism may not exclusively be a downstream consequence of Aβ accumulation or neuronal death. Mitochondrial dysfunction and cellular bioenergetics may also be implicated in AD-related encephalopathy, since decreased glucose utilization is also a consequence of mitochondrial failure [24,25]. For instance, impaired respiratory chain bioenergetics due to decreased mitochondrial function augments the processing of Aβ [26] and may favor pathological cerebral accumulation. Alternatively, the brain uses ketone bodies as an energy substrate when available [27] and brain ketone metabolism does not deteriorate in AD [15], suggesting a role for ketone-inducing neuroketotherapeutic (NKT) approaches in the treatment of AD. Additional pleiotropic neuroprotective effects have also been attributed to NKTs. Thus, dietary NKTs have gained a great deal of interest as a potential treatment for AD.

The purpose of this review was to highlight available dietary NKT approaches, the bioenergetic deficit observed in AD, putative NKT mechanisms, the current evidence for NKT in AD, and discuss a role for diet quality in NKTs.

## 2. Dietary Neuroketotherapeutic Approaches

**Figure 1:**
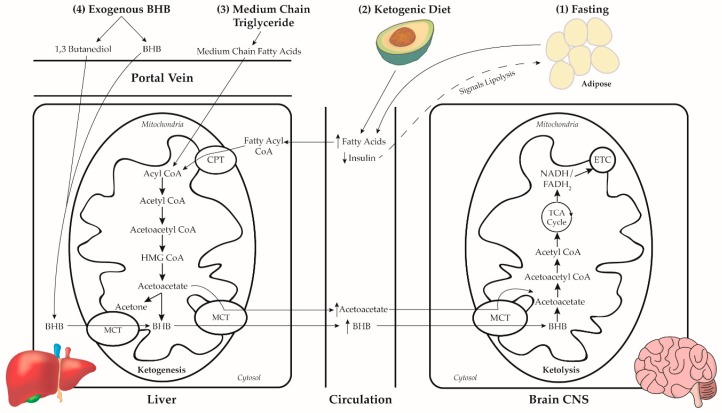
The major mechanisms to induce ketosis. **(1)** After glycogen stores are exhausted during prolonged fasting, lower levels of circulating insulin signals lipolysis of endogenous adipose, resulting in a rise in circulating free fatty acids, and ketogenesis. Fatty acids are transported into liver mitochondria for synthesis of the ketone bodies acetoacetate, β-hydroxybutyrate, and acetone to a lesser degree. Ketone bodies are transported out of the mitochondria via monocarboxylate transporters (MCT) 1 and 2, enter circulation, and enter extra-hepatic mitochondria via the same MCT 1 and 2 for ketolysis. Ketone bodies are retroconverted to acetyl CoA, enter the citric acid (TCA) cycle for ATP production, and the resulting NADH and FADH2 facilitate energy production via the electron transport chain (ETC). **(2)** The KD’s exogenous fat substrate and restricted carbohydrate initiates an action similar to fasting by increasing circulating fatty acids and reduced insulin. During a KD, both exogenous and endogenous fatty acids are destined for ketogenesis. **(3)** Medium chain triglycerides are gastrointestinally digested to medium chain fatty acids, absorb across the intestinal lumen, and rapidly enter the liver via the portal vein, bypassing the lymphatic system and peripheral circulation. Medium chain fatty acids enter the ketogenesis pathway at the level of acetyl CoA. **(4)** Exogenous β-hydroxybutyrate esters are cleaved into β-hydroxybutyrate and 1,3 butanediol by local gut esterases. Both products enter the liver via the portal vein, enter mitochondria at the β-hydroxybutyrate or acetoacetate level of ketogenesis, and enter circulation as β-hydroxybutyrate or acetoacetate.

Dietary NKTs have a history deeply rooted in the treatment of epilepsy. The first observance of NKTs are thought to date back to the fifth century BC [28]. In this study, we briefly discussed the history and physiology of four ketone-inducing approaches: prolonged fasting, the ketogenic diet (KD), MCT administration, and the administration of exogenous ketones. Figure 1 illustrates the combined pathways of ketosis through the four discussed NKTs.

### 2.1. Prolonged Fasting

Prolonged fasting has the longest and richest history of all ketone-inducing methods. Hippocrates’ classic documentation of what is thought to be a description of epilepsy in *On the Sacred Disease* eludes to the abstinence of food as a potential therapy for those afflicted with the disease [29]. This writing was in response to the overwhelming majority thought that the sacred disease was divine in nature. Hippocrates’ observations regarding fasting and diminished seizure frequency stemmed from the practice of excommunicating those stricken with the disease. Patients with epilepsy inadvertently fasted during this time with very few tools or skills to acquire food. Multiple accounts of a similar observation are found throughout the New Testament when Jesus instructed the fasting of a boy suffering from fits [30]. Successful treatment of epilepsy through prolonged fasting in the 1910s and 1920s has been widely reported in the medical literature [31,32,33,34,35,36], as it was adopted in practice by numerous physicians. Prolonged fasting is used less frequently in modern time, although the effects of alternating and intermittent variations of fasting are of interest [37] and may induce cyclical ketogenesis.

Abstinence from energy consumption, especially carbohydrate and glucogenic amino acids, stimulates peripheral lipolysis and fatty acid β-oxidation [38] in response to downregulated insulin production by pancreatic β-cells and exhaustion of stored glycogen [39,40]. Increased circulating fatty acids due to low insulin and increased glucagon are enzymatically converted to fatty acyl CoA and transported into cellular mitochondria via carntitine for β-oxidation, resulting in an increased intra-mitochondrial acetyl-CoA concentration [38,41]. Acetyl-CoA concentration saturates and exceeds the degradation capacity of the citric acid (TCA) cycle and diverts to β-hydroxy-β-methylglutaryl (HMG) CoA via HMG CoA synthase [42]. HMG CoA is cleaved by HMG CoA lyase into acetyl CoA and the ketone body, acetoacetate, which can interconvert to β-hydroxybutyrate, purported to be the most prevalent circulating ketone body, with accompanying conversion of NADH to NAD+ [43]. Although, hepatic mitochondria are the primary location for ketone body synthesis, this process is known to occur in extra-hepatic mitochondria, including astrocytes [44].

### 2.2. Ketogenic Diet

In 1921, Rollin Turner Woodyatt noted that in addition to starvation, ketone bodies were a product of “too low a proportion of carbohydrate and too high a proportion of fat” [45]. The same year, Dr. Russell Wilder reported that the KD successfully improved seizure control in 3 epileptic patients [46] and had potential for longer adherence, overcoming the disadvantage of true starvation during long-term fasting [47]. Over the following two decades, the KD was commonly used in the treatment of epilepsy but soon declined to trivial utilization in the advent of antiepileptic drugs (AED) in the late 1930s and 1940s [48]. Interest revived in the mid-1990s after Charlie Abrahams, son of movie director Jim Abrahams, succeeded in using the KD to treat his epilepsy, which was widely reported in the news media and adapted into a high-profile film. Since, the scientific literature has experienced massive growth in the report on the KD as a therapy in epilepsy and has been established as a more mainline treatment in clinics worldwide. Researchers also have growing interest in use of the KD as a therapy in neurodegenerative and metabolic conditions [49], cancer [50], and even as a performance booster in athletics [51]. 

The KD is a high-fat carbohydrate-restricted diet that induces ketone body production by mimicking fasting, a mechanism described in the previous section. The KD does not require abstinence from food for ketone body synthesis, which has the benefit of providing exogenous energy substrate, essentially reducing the necessity of converting endogenous energy stores to substrate for cellular bioenergetics. The approximate proportion of energy from the three major macronutrients in common iterations of the KD is reported in Table 1. The carbohydrate restriction of a KD prevents post-prandial rise in circulating insulin, in turn signaling the upregulation of carnitine palmitoyltransferase (CPT) to facilitate the translocation of fatty acids into the mitochondria for β-oxidation and ketone body synthesis. Both exogenously- and endogenously derived fatty acids are preferentially destined for this purpose. It appears that the concentration of circulating ketone bodies can be potentiated both through increasing fat intake (i.e., providing more of the substrate directly converted to ketone bodies) and reduced carbohydrate intake (i.e., reducing energy demand met through glycolytic metabolic processes, reduced insulin signaling, and increased demand for ketone bodies to meet bioenergetic need).

The KD is unique in that it was named for induction of its resultant energy substrate. Diets that contain carbohydrate are not referred to as the “glucogenic diet.” In contrast, there are many variations of diets that induce glucose energy metabolism and those diets or eating patterns are typically named for their food composition and carry a connotation that is generally accepted as either healthy or unhealthy. The KD has now reached a point where it is important to not only identify it by its macronutrient composition, but also the foods that compose the KD pattern. For example, some researchers and practitioners have called for using “well-formulated” versions of the diet [52] and recent evidence has pointed to an overall improvement in nutrient intake as a result of converting from a normal diet to a KD [53]. We made the case for a focus on diet quality within the KD neurotherapeutic approach.

### 2.3. Medium Chain Triglycerides

Medium chain fatty acids (MCFA) are found exclusively in coconut, palm kernel, and the milk of mammalian species [54,55,56]. Human milk is composed of approximately 10% of its energy from fat as MCFA [57] and induces a sustained mild ketosis in infants [58,59] to support energy metabolism and brain development [59,60]. Medium chain triglycerides (MCT), triglycerides containing 3 MCFA (commonly of 8 and 10 carbon chain lengths), are esterified from hydrolyzed coconut and palm kernel MCFAs [61,62], which can be consumed as supplemental MCT oil. The earliest studies of MCTs included their effect on circulating lipids [63,64] and benefit in fat malabsorptive disorders [65,66] due to their unique absorptive physiology relative to long-chain fatty acids (LCFA).

MCT supplementation is useful due to its ketogenic properties in the absence of a KD [67,68]. Huttenlocher noted that although effective in the treatment in epilepsy, the KD was severely restrictive and difficult to adhere to at times [69]. In response, his team developed the MCT diet, a version of the KD less restrictive of carbohydrate and protein than the classical KD and more ketogenic than MCT supplementation alone. The composition of the MCT diet consists of 61% energy derived from MCT, 11% from other dietary fats, 19% from carbohydrate, and 10% from protein. The high MCT intake of this diet was found to be unpalatable with significant gastrointestinal symptoms, thus, a modified MCT diet comprised of 30% of energy from MCT and 30% from other dietary fats was considered more desirable [70,71]. Clinical use of an MCT-supplemented KD have been used with varying KD ratios and contribution of energy from MCT [72].

The physiological study of the absorption of MCT dates back to the early 1950s [73]. Significantly more ketogenic than LCFA [67,68,74], the absorption and metabolism of MCT differ from long-chain triglyceride (LCT) in three ways:

(1) MCT is hydrolyzed in the gut by pancreatic lipase to their constituent fatty acids (FA) much more rapidly and completely than LCT [75]. Due to this hydrolysis action, MCT is primarily absorbed in the gut as MCFA while LCT is primarily absorbed as monoglyceride and, to a lesser extent, diglyceride and LCFA [76].
(2) MCFA is absorbed across the enterocyte and enters the portal vein for direct hepatic access [61]. In contrast, LCFA is emulsified by bile acids and packaged in micelles for absorption across the enterocyte where they are incorporated into chylomicrons and enter the lymphatic system before entering circulation [77].
(3) Once in the liver, MCFA freely enters the mitochondria for rapid β-oxidation to acetyl CoA [61]. LCFA in the cytoplasm of the hepatocytes is converted to long-chain fatty acyl CoA (LCFAcyl-CoA) [76]. The entry of LCFAcyl-CoA into the mitochondria is facilitated by binding to carnitine, where carnitine is unbound and the LCFAcyl-CoA undergoes β-oxidation. Because MCFA is absorbed at the same rate as glucose [78] and has a rapid rate of β-oxidation, the ingestion of MCT is effective at inducing ketosis.

### 2.4. Exogenous Ketones

The induction of ketosis through exogenous sources was studied as early as 1928 [79], however the administration of exogenous ketones in humans has a relatively modern history. In 1971, serving as preliminary evidence for modern ketone ester (KE) formulations, Mehlman et al. [80] and Tate et al. [81] demonstrated 1,3-Butanediol’s ketogenic metabolic fate in rats. Like MCT supplementation, exogenous ketones induced ketogenesis in the absence of a KD [82] with current potential therapeutic interest residing in the oral consumption of either ketone salts (KS) or KE. In humans, KS has been purported to modestly raise serum BHB levels [83] but may have tolerability concerns [84,85]. Ketone monoesters (KME) are purportedly more ketogenic and better tolerated than KS [83,86,87,88,89]. Although there is interest in the physiology and pharmacokinetics of ketone diesters (KDE) [90,91], current exogenous ketone products are predominantly KME, thus we will primarily discuss KME in this review.

Consumed KE is hydrolyzed by intestinal esterase to constituent R-1,3-Butanediol and BHB [86]. Both constituent products enter the liver via the portal vein, where R-1,3-Butanediol is converted to BHB and AcAc via hepatic alcohol and aldehyde dehydrogenase [92]. The resultant AcAc is converted to BHB in the hepatic mitochondria, where it is combined with the absorbed BHB and released into the circulatory system for cellular bioenergetics.

## 3. Bioenergetic Deficit in AD

Over a century after the first description of AD by Alois Alzheimer [93], the etiology of AD is still not fully understood. Decades of research have implicated cerebral changes in the pathology of AD, yet currently, approved treatments remain ineffective and investigational medications rarely show any benefit [94]. In addition to changes in cognition, hallmarks of AD include the extracellular accumulation of the aberrant protein Aβ and intracellular tau neurofibrillary tangles [95]. Although not a recent discovery, impaired brain energy metabolism has been increasingly recognized as a hallmark of the disease.

Pathophysiological changes related to AD begin in patients before clinical diagnosis of AD or its prodrome, mild cognitive impairment (MCI) [96]. It has been widely accepted that deficiencies in brain energy metabolism are observed early in AD and occur prior to symptomatic onset. Mitochondria, the cell’s respiration and energy production organelles, in the brains of patients with AD, were first shown to appear altered nearly 50 years ago [97,98]. Early studies using arterio-venous difference methods showed gross reduction of glucose uptake in the brains of AD patients [99] and, to a lesser degree, in normal aging [100]. More recently, the less invasive positron emission tomography (PET) imaging of fluorodeoxyglucose (FDG) uptake, a tracer used for the measure of tissue glucose uptake, confirms that brain glucose metabolism impairment exists in AD and in asymptomatic individuals with an elevated AD risk [8,9,10,11,12,13,14,15,16,17,18,19]. In those with presenilin 1 (PS1) mutation, amyloid precursor protein (APP) mutation, and ApoE4 allele(s), AD genetic risk factors known to affect Aβ processing, cerebral glucose hypometabolism is present prior to AD symptoms [12,13,101]. Diminishing the glucose supply in APP transgenic mice has been shown to drive the overproduction of Aβ [102]. At this time, it is unclear whether Aβ drives brain hypometabolism, vice versa, or both. The genetic risk factors for AD and Aβ aggregation exist throughout life, yet patients remain asymptomatic for at least 2 to 3 decades if they develop MCI or AD at all. Poor cellular bioenergetics and brain hypometabolism could exacerbate the genetic predisposition for cerebral protein aggregation. In patients presenting with AD, regional metabolic reduction may be as high as 33% relative to age-matched older adults [15]. Regardless of whether bioenergetic deficit is a culprit or a consequence in AD etiology, therapies targeting this dysfunction may have a role in modifying disease trajectory in patients [103].

The observance of global and regional deficit of brain glucose metabolism has been interpreted one of two ways: (1) prerequisite neuronal death and decreased synaptic activity in AD decreases glucose demand [104] or (2) due to upstream metabolic perturbation or cascade of perturbations, glucose is no longer the brain’s preferred fuel [22,105]. Despite early research demonstrating functional ketone metabolism in AD and dementia [99,106,107], the leading argument had been the former, rather than the latter. Since, key studies using PET and carbon-11 acetoacetate (^11^C-AcAc) tracer to measure cerebral ketone uptake have shown that ketone metabolism remains intact in AD [15] and augments with increasing ketone substrate availability [108,109,110]. This encouraging evidence makes a case for the latter explanation and points to the potential for dietary NKTs to serve as a therapy to target the bioenergetic crisis involved in the pathology of AD.

## 4. Putative Ketotherapeutic Benefits are Multi-Mechanistic

In addition to serving as an alternative energy substrate for glucose and potentially compensating for impaired brain glucose metabolism, NKTs have additional putative benefits that are particularly relevant to AD pathology. Ketosis may improve neuronal function, regulate neurotransmission, reduce reactive oxygen species (ROS), and modulate inflammation pathways.

Ketone-inducing therapies have been shown to influence mitochondrial function. A leading etiological hypothesis of AD implicates dysfunctional mitochondria as a causal mechanism [22,23], thus, dietary NKT approaches may modify AD at the mitochondrial level. ROS are cellular damaging mitochondrial byproducts as a result of electron loss during respiratory metabolism. The metabolism of ketones produces less ROS than glucose metabolism [111,112]. Ketosis induced by ketone monoester, caloric restriction, or the KD increases uncoupling proteins (UCP) 2, 4, and 5 of the electron transport chain in the brain [113,114,115] and UCP 1 in brown adipose tissue [116]. UCP upregulation enhances mitochondrial bioenergetics by reducing inhibitors of complex I and II of the respiratory chain [117,118,119,120,121], increasing ATP production [122,123]. Together with BHB’s suppression of glutamate transport [124], increased ATP production may balance neurotransmitter levels [125] and improve gamma-Aminobutyric acid (GABAergic) activity to regulate neuronal excitability [112,126,127]. Furthermore, upregulated UCP may attenuate matrix hyperpolarization via proton leakage to effectively reduce ROS production [128,129,130]. The KD also reduces ROS through upregulation of the antioxidants mitochondrial manganese superoxide dismutase (mnSOD) [131,132] and glutathione [133] and also acts as a powerful activator of the antioxidant and inflammation regulator, nuclear factor-E2 related factor 2 (Nrf2) [134,135]. Ketone-related mitigation of ROS may reduce mitochondrial damage through the inhibition of the mitochondrial permeability transition (mPT) pore [136,137] disallowing entry of ROS particles smaller than 1500 Daltons [138]. Although mechanistically unclear, the KD is also purported to increase hippocampal mitochondria expression [139].

Ketone bodies also have direct and indirect signaling properties that regulate neuroinflammation. BHB activates hydrocarboxylic acid receptor (HCA) 2 at 0.7 mmol/L, levels similar to those observed in human KD therapy trials [140,141], which may exert a neuroprotective effect through several downstream mechanisms [142,143,144]. Elevated BHB also inhibits histone deacetylases (HDAC) to upregulate the expression of detoxifying genes [131,132], potentially alter impaired chromatin structure and accessibility [145] and reduce cognitive deficit in an AD mouse model [146]. Ketosis and downstream HDAC inhibition upregulates the expression of brain-derived neurotrophic factor (BDNF) [147,148,149], a ligand responsible for the activation of multiple proteins involved with neuronal biogenesis and cognition [150,151].

It has recently been suggested that the gut microbiome mediates the KD’s effect on the previously discussed inflammatory and GABAergic mechanisms [152]. KD-induced changes in the microbiome may also modulate cerebral blood flow (CBF) and upregulate Aβ clearance [153].

## 5. Evidence in MCI and AD from Animal and Human Studies

For a review of the literature on NKTs in MCI and AD, we included original research and systematic reviews from PubMed and Google Scholar. We used combinations of the following search terms: “neuroketotherapeutic”, “ketone”, “fasting”, “ketogenic diet”, “medium chain triglyceride”, “MCT”, “exogenous ketone”, “ketone monoester”, “ketone diester”, “beta-hydroxybutyrate”, “BHB”, “acetoacetate”, “AcAc”, “coconut oil”, “Alzheimer disease”, “mild cognitive impairment”, “amyloid”, and “tau.” In order to identify ongoing clinical trials and potentially unreported or negative clinical trials, we also searched www.clinicaltrials.gov using the same search terms. Studies are reported in chronological order within their respective NKT classification. Considering the limited number of NKT studies within the context of AD, we included all the reported trials in either English or Spanish.

### 5.1. Animal Models

NKT studies in the transgenic mouse model of AD are minimal, almost exclusively focusing on Aβ pathology and change in neurocognition. A 43-day KD reduced Aβ brain expression relative to mice following a standard chow diet [154]. Another study evaluated the effects of 8-month administration of a ketone ester-containing diet versus a high carbohydrate diet on cognition, anxiety, and tau accumulation [155]. Both diets were similar in energy and nutrient content, except the ketone ester diet contained 21.5 g of ketone ester with 43.5% of energy from carbohydrate and the high carbohydrate diet contained 64.9% of energy from carbohydrate. The ketone ester mice exhibited better memory and less anxiety throughout and had lower brain accumulation of tau and Aβ. Non-dietary administration of ketones (600 mg injection) for 2 months improved cognition and protected against Aβ neurotoxicity relative to mice receiving a normal saline injection [156]. Another study demonstrated that a KD approach improved motor performance but not memory at 12 weeks and had no effect on tau or Aβ deposition at 16 weeks [157]. Similarly, 1-month administration of a KD did improve motor performance and had no effect on cerebral Aβ [158]. Although not in a transgenic mouse model, a 1-month KD elicited change in hippocampal expression of metabolism-related genes [159], a region particularly affected by atrophy in AD.

### 5.2. Humans

Evidence for NKT in AD is very preliminary, however promising. To date, there have been various reports on MCT treatment, one report on ketone ester treatment, three trials reported on KD interventions, and no reports on prolonged fasting. It should be noted that prolonged fasting is not a likely NKT candidate in AD due to the risk of decreased nutrition status in the elderly and AD [160]. A summary of all included human trials is included in Supplementary Table S1.

#### 5.2.1. MCT Treatment

Within the context of AD, MCT treatments as an NKT approach hold the most evidence. Most are small preliminary studies that have generally demonstrated modest cognitive benefit and investigated potential effects on additional biomarkers of brain metabolism.

In a preliminary study to test beta-hydroxybutyrate’s clinical potential in AD, Reger et al. enrolled 20 patients with probable AD or MCI and, in a random order across two visits, were given an MCT blend (40 mL of MCT blended with 152 mL of whipped cream) or 232 mL of whipped cream alone [161]. BHB concentration increased to 0.54 mmol/L 90 minutes post consumption of the MCT treatment and correlated with improvement in paragraph recall test scores from the timepoint. Apo E4+ carriers had a higher BHB production in response to the MCT treatment, yet the Apo E4- subjects had larger improvement in cognition.

In one of the earliest and the largest MCT trial in AD to date, Henderson et al. administered caprylic triglyceride (C8:0 MCT referred to as AC-1202) treatment or placebo to 152 AD subjects [162]. Two hours after AC-1202 consumption, beta-hydroxybutyrate levels showed an approximate four-fold increase. On study day 90, serum levels rose from about 0.1 mM to 0.40 mM. On day 45, the active treatment group’s Alzheimer’s Disease Assessment Scale-Cognition Subtest (ADASCog) score was significantly better than the placebo group’s score, and among APOE4 negative subjects, this advantage was also observed at 90 days. Based on this study, the Food and Drug Administration classified AC-1202 as a medical food that could be used for the “dietary management of metabolic processes associated with mild to moderate Alzheimer’s disease”. It is currently marketed under the brand name Axona [163].

Maynard and Gelblum reported a retrospective chart review of 55 patients with probable mild to moderate AD with documentation of having received Axona treatment for 6 months or longer [164]. Over a mean follow-up of 18.8 months, 79.5% of patients exhibited improvement or stability of symptoms. Nearly half of patients had available baseline and during-treatment MMSE scores, which remained stable during the course of the treatment.

Farah reported a case-study of C8:0 MCT intake by a 70-year-old male with mild AD [165]. The patient gradually titrated C8:0 dosage to 20 g over 7 days and maintained that dosage for 102 additional days. Over that time, his MMSE and MoCA scores improved from 23 to 28 and 24 to 28. Baseline FDG PET scans revealed regional glucose hypometabolism which did not change due to the MCT treatment.

Rebello et al. conducted a trial with six participants with MCI [166]. The participants consumed either 56g of MCT oil (C8:0 and C10:0) or placebo (canola oil) within 6 oz. of fruited yogurt for 24 weeks. Two participants withdrew from the study and recruiting woes were noted. Half of the remaining participants received the treatment (one each ApoE4+ and ApoE4-) and the other half, the placebo. After 24 weeks, the ApoE4- patient demonstrated improved word recall, word recognition, and remembering with overall improvement on the ADASCog and the ApoE4 homozygous patient had improved word recall, word recognition, and remembering but decreased overall ADASCog performance.

Croteau et al. enrolled 15 patients with possible or probable AD and investigated the effect of two different emulsified MCT supplements (1: 55% C8:0 + 35% C10:0 and 2: 100% C8:0) on PET-derived brain ketone (via 11C-acetoacetate) and glucose (18F-fluorodeoxyglucose) uptake [109]. Eleven participants completed the study protocol to consume 30 g of MCT per day for one month. Post intervention, cerebral uptake of glucose was unchanged, however, both global and regional cerebral ketone uptake increased significantly and correlated with plasma ketone concentration due to both MCT interventions. 

A novel biomarker of interest in AD is CBF, as it is purported to decrease in patients with MCI and AD and likely relates to the hypometabolic state observed in AD [167]. Torosyan et al. enrolled 16 participants with mild to moderate AD to a double-blind, placebo-controlled trial to test Axona’s effect on CBF [168]. Fourteen participants received the 40 g Axona (20 g MCT as C8:0) treatment and two participants received the placebo. CBF was measured at baseline prior to a dose of either the treatment or placebo and 90 min post dosage to measurement of Axona’s acute effects. To measure long-term effects, participants continued the treatment/placebo protocol for 45 days, at which point their CBF was measured again prior to dosage and 90 min post daily dose. In the entire group, Axona elicited no change in CBF either acutely or long-term. Accounting for ApoE4 status, those negative for ApoE4 increased regional CBF in the superior lateral temporal cortex after 45 days of treatment with Axona, yet the ApoE4+ carriers exhibited no change in regional or global CBF.

Ota et al. reported no acute effect of MCT on cognition after 20 patients with mild to moderate AD had no improvement in cognition 2 h after consuming a 20-g emulsified MCT dosage [169]. The research team followed this acute study with a 12-week open label clinical trial in 19 of the 20 patients from the first study. The protocol required that patients consume the same 20-g MCT dosage each day with meals. In 16 completers, patients demonstrated progressive improvement in working memory, short-term memory, and processing speed at 4 weeks, 8 weeks, and 12 weeks. The findings of this study suggest that a ketone-body inducing agent, in the absence of dietary changes, does not provide acute cognitive benefit, but may elicit physiological improvement in AD.

Fortier et al. randomized 52 MCI patients ≥55 years old 1:1 to either 30 g of proprietary MCT (2x per day of 125 mL liquid containing 60% C8 and 40% C10 saturated fatty acids) or placebo (2x per day of 125 mL liquid containing high oleic sunflower oil) for 6 months [110]. The intervention had 75% compliance with *n* = 8 withdrawals from the intervention group and *n* = 6 withdrawals from the placebo. The intervention group completers consumed 90% of planned MCT intake for the duration of the study. Global CMR_Ketones_ increased by 230% in the intervention group (increase from 1.1 μmol/100 g/min to 2.5 μmol/100 g/min) and similarly across six regions of interest, remaining unchanged in the placebo group. Accounting for a combined value of CMR_Glu_ and CMR_Ketones_, this equated to a 3.6% global increase in brain energy uptake in the intervention group. Furthermore, the intervention group experienced statistically significant improvement in free recall, visual memory, inhibitory capacity, and visual selective attention. Change in scores on the Trail Making–Visual Scan, Boston Naming Test and Verbal Fluency were positively correlated with plasma ketone concentration and change in scores from the Trail Making–Visual Scan and composite z-score of processing speed were positively correlated with CMR_Ketones_.

#### 5.2.2. Coconut Oil

Coconut oil was included in this review as its use has grown in interest within the context of therapeutics in AD. Coconut oil possesses a high MCT content, comprised of approximately 9% C8:0, 7% C10:0, and 47% C12:0 as a percentage of total fatty acid content [170]. Since C12:0 is less ketogenic than C8:0 and C10:0 [171], coconut oil must be taken in higher doses to elicit a ketogenic response. Previous evidence suggests that 40 mL (2–20 mL doses) of coconut oil was required to modestly raise serum ketone levels [172], thus, it is a very mild NKT in appropriate doses. At this time, there is very little evidence surrounding the use of coconut oil in MCI or AD.

Yang et al. randomized 44 institutionalized patients with very mild to severe AD [173]. Patients were randomized 1:1 to receive similar diets with either the addition of 40 mL of extra virgin coconut oil or no coconut oil for 21 days. At the end of the intervention, the coconut oil group’s MEC-Lobo (a Spanish language cognitive assessment similar to the MMSE in English) improved 4.5 points and the control group’s scores did not change from baseline.

Chan et al. randomized 40 participants with mild to severe AD 1:1 to either the intervention of 60 mL of cold pressed virgin coconut oil or placebo of water containing coconut essence [174]. The study had a high dropout rate of six participants from the placebo and 12 from the intervention, primarily due to diarrhea, abdominal discomfort, and smell or taste of the coconut products. Limitations due to the nearly 50% dropout rate exist, yet the authors reported no significant cognition-related findings from this study.

De la Rubia Ortí equally randomized 44 institutionalized patients with moderate to severe AD to isocaloric Mediterranean diets with either 40 mL of unspecified coconut oil or no coconut oil for 21 days [175]. The group that received coconut oil in addition to the Mediterranean diet improved episodic memory, temporal orientation, and semantic memory from baseline. The control group had no change in cognitive test performance. Interestingly, the authors observed a more robust cognitive response to the intervention in females with moderate AD, although improvements were observed in both sexes across the spectrum of AD severity. Dietary intake was not reported in this study, but Mediterranean diets are commonly associated with high diet quality, thus, this study ties into the following discussion in this review regarding diet quality and NKTs.

#### 5.2.3. Exogenous Ketone Treatment

To date, the only evidence for a ketone ester treatment in AD is a case-study by Newport et al. [176]. The report entailed early MCT treatment and transition to ketone monoester treatment alone in a 63-year-old Caucasian male patient with sporadic AD. The patient was positive for ApoE4 and had seen continuous decline in cognition over the course of 12 years. Seventy-five days after the initiation of a ketogenic formula (165 mL/daily of a 4:3 mixture of MCT and coconut oil), the patient’s MMSE score improved 8 points from 12 to 20. After 20 months of treatment with the ketogenic formula, the patient’s mood and demeaner was reported to have improved and, objectively, his ADASCog score improved by 6 points and his ability to carry out activities of daily living improved by 14 points. Furthermore, after several years of documented brain volume degradation by MRI, the patient’s MRI remained stable after starting the treatment. After involvement in a research study of an investigational AD drug now known to have caused patients to decline rapidly [177], the patient’s disposition deteriorated. The primary purpose of this report was to demonstrate that the consumption of ketone monoesters is capable of elevating circulating ketone bodies. The patient initiated a ketone monoester supplement of 21.5 g 3x per day, gradually increasing to a dosage of 28.7 g 3x per day. Serum BHB levels increased acutely in a dose-response fashion after ingestion of the ketone monoester supplement, and, although cognition was not objectively measured at this point, the patient’s disposition was reported to improve, as he had regained some recollection and ability to perform activities of daily living. 

#### 5.2.4. Ketogenic Diet

Human investigation of the KD as an NKT for AD is limited, yet three positive studies are documented, two of which were reported in the last 2 years.

Krikorian et al. randomized 23 patients with MCI to either a low-carbohydrate (5–10% of energy, *n* = 12) or high carbohydrate (50% of energy) dietary intervention for 6 weeks [178]. Essentially, the low-carbohydrate group achieved a low-carbohydrate calorie restricted diet by successfully reducing carbohydrate intake (190 g ± 56 to 34g ± 14) and total energy intake (1762 ± 481 to 1042 ± 347). The high carbohydrate group had no significant changes in energy and macronutrient intake. Within the low-carbohydrate group, there was observance of trace urinary ketosis (5.4 mg/dL), reduced fasting insulin concentration, and improved verbal memory scores from baseline to week 6.

Our group was the first to evaluate the feasibility and preliminary efficacy of a 3-month KD intervention in patients diagnosed with AD [140]. Furthermore, this particular intervention is the only human KD intervention study within any condition to report extensive actual dietary intake changes due to KD intervention [53]. The focus of the dietary intervention was to achieve a “well-formulated” 1:1 KD ratio (energy as: 70–75% fat, 5–10% carbohydrate, and 20–25% protein) with a high intake of non-starchy vegetables, avocados, and nuts and seeds. Participants also consumed between 1 and 2 tablespoons of MCT oil daily. Ten of the 15 participants enrolled in the study consumed a nutrient rich KD with a KD ratio of 1.23:1, achieved urinary and serum ketosis, and improved ADAS-Cog scores by an average of 4.1 points from baseline to month 3. Excluding one protocol-non-compliant participant for self-discontinuation of medication during the intervention, protocol-compliant participants had an ADAS-Cog mean improvement of 5.3 points. After a 1-month suspension of the KD, the mean ADAS-Cog score regressed to baseline level.

Brandt et al. recently reported preliminary findings from 14 study-completing participants in a 27-person, 12-week feasibility trial of the Modified Atkins Diet in patients with MCI and early AD [141]. The report included nine participants that were randomized to MAD and five participants randomized to the National Institutes of Aging’s recommendations for senior nutrition (NIA diet). Intent to treat analysis showed a slight trend toward composite memory improvement in the MAD group and a slight decline in the NIA diet group. Within the MAD group alone, the results were further analyzed using two different definitions of MAD adherence: (1) at least trace urinary ketone production at one or more study visits (*n* = 5 adherent and *n* = 4 non-adherent) and (2) self-reported consumption of ≤35 g of carbohydrate (*n* = 6 adherent and *n* = 3 non-adherent). Participants positive for ketosis at week 6 showed significantly improved composite memory scores at the same timepoint from baseline, while the MAD non-adherent individuals showed decline in composite memory. At 12 weeks, both groups had slight non-significant improvement in composite memory. Using the carbohydrate restriction definition of ≤35 g for MAD adherence, composite memory scores had a trending modest improvement at week 6 in the MAD adherent group and slight decline in the non-adherent group. Regardless of method for compliance determination, this study also reported that MAD adherent individuals had increased energy levels from baseline to week 6 assessment.

#### 5.2.5. Limitations

It should be noted that the literature on NKTs in MCI and AD is currently limited. Many of the studies had small sample sizes and some had large dropout rates. Many studies did allow for sufficient time between cognitive testing to mitigate test-retest artifact, however, studies with fewer than 4 weeks of time allowance between repeated cognitive measures are a risk of this artifact. From the perspective of diet quality within NKT interventions, data is very limited at this time due to a lack of dietary intake report. It is also impossible to know whether unreported trials with negative findings exist.

#### 5.2.6. Ongoing Clinical Trials

There is compelling preliminary evidence to support the use of dietary NKT approaches in AD. With great interest in this subject, we highly anticipate the results of multiple ongoing clinical trials to assess the efficacy of different NKTs in AD. An RCT at the University of British Columbia (NCT02912963) is testing biomarker response to dose-dependent MCT treatment or high oleic sunflower oil (5 dosing groups: 10 g, 20 g, 30 g, 40 g, or 50 g) for 10 days in 40 patients with mild to moderate AD. Outcomes include adverse events, depth of ketosis, CBF, brain metabolite spectroscopy, glucose response, and change in activity level. The group at Johns Hopkins University (NCT02521818) has already reported preliminary evidence from their RCT investigating the feasibility of their Modified Atkins Diet [179] vs. the NIA Diet for Seniors and their effects on the cognition and activities of daily living. We await their full report with 27 patients with MCI and probable AD. Furthermore, there are two large RCTs testing outcomes related to the consumption of a well-formulated KD in MCI and AD. A group from Wake Forest (NCT03472664) is testing the effectiveness of a 4-month “Modified Mediterranean KD”, a KD rich in olive oil, fatty fish, and non-starchy vegetables with <20 g carbohydrate restriction, vs. a low-fat diet at altering cerebral spinal fluid concentration of Aβ, cognition, and cerebral blood flow in 120 patients with MCI and AD. Our group (NCT03860792) is currently conducting an 80-person efficacy trial of our previous 3-month KD [53,140] with prescriptive non-starchy vegetable, fatty fruit, MCT and olive oil, and fatty fish intake vs. the therapeutic lifestyle changes (TLC) diet [180] in AD. Our outcomes include change in cognition, brain metabolism, and mitochondrial function. Collectively, these studies will contribute to a broader understanding of the effectiveness of NKT approaches in AD and, importantly, establish more evidence for the potential role of KDs with high diet quality in synergizing benefits of these therapies.

## 6. Role of Diet Quality in NKTs

Consuming a high-quality nutrient-rich diet is important for long-term health [181]. Criticism of NKT approaches, primarily the KD, hinges on the argument of their poor reputation of low adherence, unsustainability, and suspected unhealthfulness for being high in fat and low in nutrient-dense foods [182,183,184]. While these arguments are justified in the absence of evidence to the contrary, our group demonstrated that patients with AD could consume and tolerate a nutrient-dense KD for 3 months with a non-starchy vegetable intake as high as 7 servings/day [53]. This diet successfully induced robust ketosis. A well-formulated KD with focus on non-starchy vegetable and quality fat intake [185] was also feasible in 194 patients with type 2 diabetes for 2 years [186]. In this study, elevated serum BHB levels were maintained, reaching or exceeding the goal of 0.5 mmol/L in nearly 33% of cases, and a myriad of health-related benefits were observed.

Concerns regarding NKT compliance and adverse health outcomes stem from, prior to recent history, exclusive investigation in epilepsy. To maintain seizure control, strict classical KD ratios (i.e., 4:1 or 3:1) were historically implemented. There is very little flexibility built into these KD iterations, which, indeed, inherently restricts many nutrient-rich food components. In this regard, KD research and clinical use has been merely defined by its macronutrient profile. At this point, this is partially justifiable as most research in many neurodegenerative disorders is in the feasibility and proof of principle stage. However, the theory of beneficence from the presence of ketone bodies as an alternative brain fuel and mounting evidence that ketones can be generated in less restrictive NKT approaches opens a door to identify whether diet quality influences NKT efficacy, adherence, and sustainability.

### 6.1. Components of a High-Quality KD

By its very definition, the KD’s ultimate goal is to achieve nutritional ketosis. To attain this, the diet must adequately restrict carbohydrate with a majority of energy derived from dietary fat. While improved cognition coincided with a serum BHB level as low as 0.3 mmol/L [140], it has been suggested that target nutritional ketosis should be ≥0.5 mmol/L [52,185,186]. These levels can be attained or exceeded following a 1:1 KD ratio, which, on a 2000 kcal diet, equates to 200 g of fat and 50 g of carbohydrate intake per day. The remainder of energy should be consumed as protein to maintain lean body mass and function, approximately 100 grams per day in the 2000 kcal example. Stricter KD ratios may result in more robust ketosis.

Due to the requisite increase in fat intake, attention to fat source should be a primary focus. Fatty acid composition in the general diet and related health outcomes have been studied extensively and remain controversial [187,188,189]. Within the context of a carbohydrate-restricted diet or KD, diets comprised of either majority SFA or PUFA are both ketogenic without negative change in circulating LDL cholesterol, SFA, or inflammatory markers [190,191,192]. The role of SFA in a ketogenic diet may be altered relative to carbohydrate-rich diets as SFAs are highly ketogenic with a primary metabolic fate as energy substrate [193]. It is also important to consider unsaturated fat, MUFA and PUFA intake. MUFAs are linked to a wide array of health benefits, including reduced inflammation and decreased risk of type 2 diabetes and cardiovascular disease [194], especially when derived from plant sources [195]. PUFAs are divided into two main essential groups, omega-3 and -6, depending on the location of their point of unsaturation. Omega-3s have been shown to be anti-inflammatory [196], whereas omega-6s are thought to be more proinflammatory [197]. As one of the putative benefits of ketone bodies may be attributable to favorable changes in inflammation, a high omega-3/omega-6 ratio may extend NKT benefits through potentiation of PPAR activation [198]. All considered, it is advisable that the fat content of high quality KDs comprise of mostly whole foods that provide a majority MUFA, omega-3 PUFA, and SFA. Favorable sources for each of the FA categories are detailed in Table 2.

High-quality KDs should be rich in non-starchy vegetables [199]. The most healthful diets generally attribute many of their healthy characteristics to a high fruit and vegetable composition [200], particularly dark green and brightly colored vegetables [201,202]. Ideal components of a carbohydrate-restricted diet, non-starchy vegetables are nutrient-dense, low in carbohydrate, and can be consumed in high volume, which may be satiating. Non-starchy vegetables may influence the efficacy of the KD due to their antioxidative and overall health properties and may also be key to improved sustainability and tolerability of the KD. With the potential to extend the effects of ketone bodies, polyphenols found in colorful plant-based foods are protective of mitochondrial function [203,204,205]. Consuming an array of vegetables adds variety to the KD, as well as fiber which has clinical report of improving KD tolerability. Fiber also serves as an energy substrate for gut bacteria, converting ingested fiber to short chain fatty acids (SCFA) that support ketosis [206] and mitochondrial function [207]. Due to its sugar and total carbohydrate content, fruit should be restricted on the KD. The exception to this rule is avocado, which contains a very high-fat and low-net carbohydrate content as most of its carbohydrate is counted as fiber and is linked to multiple health benefits [208]. If consumption does not exceed the allotted daily carbohydrate intake level, a small portion of polyphenol-rich berries may also provide benefit within the KD [209].

Another important consideration in KD formulation is electrolyte intake. Ketogenesis through prolonged fasting and the KD is natriuretic [210]. Increased sodium excretion stimulates upregulated aldosterone expression to preserve sodium homeostasis by sodium reabsorption in the kidney [211]. Conversely, increased aldosterone stimulates potassium excretion in the urine and the release of intramuscular potassium to maintain blood concentration. The majority of symptoms cited on a KD, such as GI symptoms and muscle cramping, are attributable to this cycle, causing concern for the tolerability and sustainability of the KD. Foods that comprise a high-quality KD are naturally low in sodium, thus sodium chloride (table salt) usage is recommended to replenish ketogenesis-related sodium excretion and mitigate potassium excretion. Additional sodium consumption is indicated in the presence of natriuretic symptoms during a KD. Dark green vegetables, avocados, and mushrooms are rich in potassium, while dark greens and nuts are rich in magnesium, another indication of their importance in a high quality KD. Our data suggests that, even in a nutrient dense KD, both of these nutrients are nutrients of concern [53]. Magnesium needs can be met by consuming green leafy vegetables [212]. The additional intake of potassium by potassium containing salt may be necessary. Bone broth is commonly recommended to replenish these nutrients, however its richness in potassium is dependent upon the amount of meat included in its preparation and has negligible magnesium contribution [213].

### 6.2. Considerations of Diet Quality in Non-KD NKTs

While the potential benefits of dietary NKTs are theoretically related to ketogenesis, it is important to consider how consuming a healthy diet may potentiate these benefits. For example, the Mediterranean diet’s purported health and cognition benefits are driven by foods that comprise this diet pattern. Fruits and vegetables have polyphenols that are pertinent for mitochondrial function [203,204,205], antioxidative properties [214], and carotenoids that correlate with cognitive function [215,216]. Moreover, diets rich in olive oil are considered protective of health and cognition through many overlapping mechanisms [217]. Future study of non-KD NKTs would benefit from distinguishing whether following a conjunctive nutritionally mindful approach enhances therapeutic response. RCTs of MCT or exogenous ketones should, at minimum, include dietary collection to investigate the diet’s moderation effect. Further consideration for RCTs could include dietary protocol implementation in addition to MCT or exogenous ketone supplementation.

## 7. Conclusions

It is crucial to identify effective treatment for AD. Currently, in its early stage of investigation, NKTs offer a promising and potentially diverse approach in the treatment of AD. Currently, there is insufficient evidence to recommend NKTs for the clinical treatment of MCI or AD. Further investigation of their impact on AD and how diet quality may contribute to treatment efficacy and sustainability is needed.

## Figures and Tables

**Table 1:** Macronutrient composition of common ketogenic diet (KD) formulations ^1^.

Diet Formulation	Fat % (g)	Carbohydrate % (g)	Protein % (g)
4:1 Ketogenic Diet	90% (200)	2% (10)	8% (40)
3:1 Ketogenic Diet	87% (193)	4% (20)	9% (45)
2:1 Ketogenic Diet	82% (182)	8% (40)	10% (50)
1:1 Ketogenic Diet	70% (156)	10% (50)	20% (100)
Modifed Atkins Diet	70% (156)	5% (25)	25% (125)
MCT Diet ^2^	71% (158) ^3^	19% (95)	10% (50)

^1^ Grams of macronutrient based on 2000 kcal reference. ^2^ Medium-Chain Triglyceride Diet. ^3^ Dietary fat composed of 60% of energy from MCT oil and 11% of energy from other dietary fats.

**Table 2:** Favorable ketogenic diet fatty acid sources.

MUFA ^1^	Omega-3 PUFA ^2^	Omega-6 PUFA ^2^	SFA ^3^
Avocado	Chia Seeds	Nuts & Seeds	Butter
Lard	Fatty Fish	Dark Poultry	Coconut Oil
Nuts & Seeds	Flaxseeds	Red Meat	Eggs
Olive Oil	Walnuts	Non-Starchy Vegetables	MCT Oil
Olives			Dark Poultry
			Red Meat

^1^ Monounsaturated Fatty Acids. ^2^ Polyunsaturated Fatty Acids. ^3^ Saturated Fatty Acids.

## Supplementary Materials

The following are available online at https://www.mdpi.com/2072-6643/11/8/1910/s1, Table S1: Summary of neuroketotherapeutic studies included in this review.
