# Supplementary material for: Dietary Neuroketotherapeutics for Alzheimer’s Disease: An Evidence Update and the Potential Role for Diet Quality
Source: Nutrients. 2019 Aug 15;11(8):1910. doi: 10.3390/nu11081910 (PMC6722814; doi:10.3390/nu11081910)
Supplement: Supplementary file 1 [file nutrients-11-01910-s001.pdf]

Table S1. Summary of neuroketotherapeutic studies included in this review.

Study	Country	Design	Sample[1]	Diagnosis	Intervention	Result
**MCT Oil**						
Reger (2004)	USA	Crossover	20	Probable MCI or AD	1 dose of 40 mL MCT or placebo	Improved paragraph recall 90 minutes post-MCT.
Henderson (2009)	USA	RCT	152	Mild/Moderate AD	3 months of 20g MCT (C8) vs. placebo	Better ADASCog with MCT vs. placebo at day 45. ApoE4 negative individuals also had better ADASCog with MCT at day 90.
Maynard (2013)	USA	Chart Review	55	Probable Mild/Moderate AD	Unspecified Axona dosage for 6+ months	79.5% of patients had stability or improvement over 18.8 month follow up.
Farah (2014)	USA	Case Report	1	Mild AD	109 days of 20g MCT (C8)	5-point improvement in MMSE and 4-point improvement in MoCA. No change in glucose uptake.
Rebello (2015)	USA	RCT	4	MCI	24 weeks of 56g MCT (C8/C10) vs. placebo	Limited feasibility with high dropout and recruitment challenges. Global ADASCog scores improved in the ApoE4 negative patient and slightly worsened in the ApoE4 positive patient.
Croteau (2018)	Canada	Single-Arm Trial	11	Possible/Probable AD	1 month of 30g MCT (C8/C10) vs. 30g MCT (C8)	Increased cerebral ketone uptake (measured by PET) associated with both MCT formulations.
Torosyan (2018)	USA	RCT	16	MCI or AD	45 days of Axona [20g MCT (C8)] vs. placebo	No change in cerebral blood flow after 1 dose or at 45 days. Increased superior lateral temporal cortex blood flow in ApoE4 negative patients at day 45.
Ota (2019)	Japan	Crossover	20	Mild to Moderate AD	1 dose of 20g MCT (C8/C10) or placebo	No between group difference in cognitive performance 120 minutes post-administration of the MCT formulation or placebo.
Ota (2019)	Japan	Single-Arm Trial	16	Mild to Moderate AD	12 weeks of 20g MCT (C8/C10)	Improvement from baseline in immediate and delayed logical memory tests at 8 weeks. Improvement from baseline in digit-symbol coding and immediate logical memory tests at week 12.
Fortier (2019)	Canada	RCT	38	MCI	6 months of 30g MCT (C8/C10) vs. placebo	230% increase in cerebral ketone uptake (measured by PET) in MCT group. No change in cerebral glucose uptake. Multiple cognitive domain improvements in MCT group that positively correlated with plasma ketone levels.
**Exogenous Ketone**						
Newport (2015)	USA	Case Report	1	Early Onset Sporadic AD	8 weeks of 28.7g ketone monoester 3x/day	Acute elevation in serum BHB and observed improvement in recollection and function.
**Coconut Oil**						
Yang (2015)	Spain	RCT	44	Institutionalized AD	21 days of 40mL extra virgin coconut oil vs. control	4.5-point MEC-Lobo (validated Spanish cognitive test) improvement in coconut oil group. No cognitive change in control group.

Chan (2017)	Malaysia	RCT	22	Mild to Severe AD	6 months of 60mL cold pressed virgin coconut oil vs. placebo	High dropout as 40 participants were randomized and 18 withdrew (12 intervention, 6 control). No change in cognition in either group.
De la Rubia Ortí (2018)	Spain	RCT	44	Institutionalized AD	21 days of Mediterranean diet plus 40mL coconut oil vs. Mediterranean diet alone	The Mediterranean diet plus coconut oil group improved episodic memory, temporal orientation, and semantic memory relative to the Mediterranean diet group alone.
**Ketogenic Diet**						
Krikorian (2012)	USA	RCT	23	MCI	6 weeks of carbohydrate restriction (<10% energy) vs. high carbohydrate (~50% energy)	Trace urinary ketone production. Verbal memory improvement in the carbohydrate restriction group that correlated with urine ketone levels.
Taylor (2018)	USA	Single-Arm Trial	10	Very Mild to Moderate AD	3 months of well-formulated KD with MCT supplementation	4.1-point improvement in ADASCog scores at end of intervention. Scores returned to baseline values after 1-month discontinuance of KD.
Brandt (2019)	USA	RCT	14	MCI or AD	12 weeks of MAD vs. NIA diet for seniors	MAD group had no change in global memory. Global memory worsened in NIA group. Ketosis-producing MAD participants had improved global memory at week 6 and no change between week 6 and 12.

[1]Sample number reflects the sample size included in data analysis Abbreviations: AD, Alzheimer's disease; ADASCog, Alzheimer's Disease Assessment Scale Cognitive Subscale; BHB, beta-hydroxybutyrate; KD, ketogenic diet; MAD, Modified Atkins Diet; MCI, mild cognitive impairment; MCT, medium-chain triglyceride; MMSE, Mini-Mental State Examination; MoCA, Montreal Cognitive Assessment; NIA, National Institutes on Aging; RCT, randomized controlled trial.
